# Aging dysregulates neutrophil extracellular trap formation in response to HIV in blood and genital tissues

**DOI:** 10.3389/fimmu.2023.1256182

**Published:** 2023-11-15

**Authors:** Laura Moreno de Lara, Alexandra Werner, Anna Borchers, Francisco J. Carrillo-Salinas, Wendelin Marmol, Siddharth Parthasarathy, Vidya Iyer, Alison Vogell, Diego Illanes, Ana C. Abadía-Molina, Christina Ochsenbauer, Charles R. Wira, Marta Rodriguez-Garcia

**Affiliations:** ^1^ Department of Immunology, Tufts University School of Medicine, Boston, MA, United States; ^2^ Instituto de Biopatología y Medicina Regenerativa, Centro de Investigación Biomédica, Universidad de Granada, Granada, Spain; ^3^ Program in Genetics, Molecular, and Cellular Biology, Tufts University School of Medicine, Boston, MA, United States; ^4^ Department of Gynecology and Obstetrics, Tufts Medical Center, Boston, MA, United States; ^5^ Departamento de Bioquímica y Biología Molecular 3 e Inmunología, Universidad de Granada, Granada, Spain; ^6^ Department of Medicine and UAB Center for AIDS Research, University of Alabama at Birmingham, Birmingham, AL, United States; ^7^ Department of Microbiology and Immunology, Geisel School of Medicine at Dartmouth, Lebanon, NH, United States

**Keywords:** HIV prevention, neutrophils, aging, neutrophil extracellular traps, NETs, female genital tract, women, menopause

## Abstract

Women acquire HIV through sexual transmission, with increasing incidence in women >50 years old. Identifying protective mechanisms in the female genital tract (FGT) is important to prevent HIV-acquisition in women as they age. Human genital and blood neutrophils inactivate HIV by releasing neutrophil extracellular traps (NETs), an innate protective mechanism against HIV-infection. However, how NET formation is triggered by HIV in different tissues and whether this mechanism is affected by aging remain unknown. We demonstrate that the mechanisms that trigger NET release in response to HIV are different in blood and genital tissues, and that NET release decreases with aging. In blood neutrophils, HIV stimulation independently activated calcium pathways and endosomal TLR8, but aging reduced calcium responses, resulting in delayed NET release. In contrast, calcium responses were absent in genital neutrophils and NET release was triggered preferentially through TLR8 activation, but aging impaired this pathway. HIV induced NET formation through non-lytic pathways in blood and FGT neutrophils, except for a small subset of NETs that incorporated annexin V and lactoferrin predominantly in blood, suggesting proinflammatory and lytic NET release. Our findings demonstrate that blood neutrophils cannot model genital neutrophil responses which has important implications to understanding protection against HIV acquisition.

## Introduction

Human Immunodeficiency Virus (HIV) continues to be a global and public health problem with 38.4 million people living with HIV worldwide and 1.5 million new infections in 2021 (1, 2). Women represent 54% of people living with HIV globally but, in endemic areas such as Sub-Saharan Africa, they account for more than 60% of new HIV infections (1, 3). Furthermore, the number of new HIV infections is rising worldwide in women older than 50 years of age (4–6), representing an urgent clinical need.

Sexual transmission is the main route for HIV acquisition in women of all ages (7). However, the rate of HIV transmission per sexual act is very low (0.08%) (8), which indicates that local immune mechanisms in the female genital tract (FGT), a main portal for HIV acquisition in women, contribute to the prevention of HIV infection (9, 10). For example, barrier mechanisms including epithelial cell integrity, mucus and secretions are known to play key roles in mucosal protection (11, 12). Epidemiological studies indicate that older women have increased susceptibility to infection (13–15), and several studies have identified changes in HIV target cells that may contribute to increased susceptibility (16–18). However, how innate protective mechanisms in the FGT change with aging, remain largely unknown (18).

In the FGT, neutrophils are abundantly present under physiological conditions, accounting for 10–30% of immune cells (CD45+) (11, 19). Neutrophils display multiple innate defense mechanisms including phagocytosis, degranulation, production of reactive oxygen species (ROS), and release of Neutrophil Extracellular Traps (NETs) (9, 20). NETs are DNA fibers coated with antimicrobial proteins that neutrophils release to trap and kill pathogens (20). NET release has been described in response to sexual transmitted infections (STIs), such as *Neisseria gonorrhoeae* (21). However, neutrophil responses have shown a controversial role in HIV infection. In chronic HIV/SIV infection, detrimental effects of NETs have been reported, such as the destruction of immune cells, inflammation and tissue damage that would contribute to the pathology observed in chronic infection (22, 23). In addition, in the context of genital inflammation and STIs, neutrophil-derived molecules in cervico-vaginal secretions were associated with increased risk of HIV acquisition (24–26). In contrast, other studies support a role for neutrophils in protection against HIV acquisition. A recent study reported that genital neutrophils contribute little to tissue remodeling under physiological conditions, challenging the notion that tissue neutrophils are involved in tissue damage in the absence of pathological inflammatory stimuli (27). Additionally, clinical evidence supports a protective role in neutrophils in response to HIV: some studies in Africa found associations between low neutrophil counts in blood (neutropenia) and higher risk of HIV acquisition in sex workers (28), and higher risk of HIV intrauterine transmission (29). These apparently contradictory findings show a gap in knowledge about neutrophil responses to HIV and the need to determine the role of NETs in HIV acquisition in the genital mucosa. Importantly, two main types of NET release have been described: lytic NET release, a ROS-dependent mechanism of late NET formation mediated through NADPH oxidase (NOX2) that involves cell membrane disruption; and non-lytic NET release, a rapid, NOX2-independent and intracellular calcium-dependent mechanism in which NETs are extruded, but neutrophils remain viable (30–35). Blood neutrophils have the ability to release NETs in response to HIV (36–38). Further, we recently demonstrated that neutrophils from the female genital mucosa release NETs immediately after HIV stimulation (within minutes) in premenopausal women, and NETs irreversibly inactivate HIV to prevent infection of target cells (37). We and others demonstrated previously that HIV-induced NET release was partly dependent on HIV-RNA recognition through Toll-like receptor (TLR) 7/8 pathways and ROS production at late time points after viral stimulation in blood (36) and genital neutrophils from premenopausal women (37). However, the underlying mechanisms responsible for triggering NET release immediately after HIV stimulation remain undefined. Furthermore, whether this anti-HIV mechanism is modified as women age remains a gap in our knowledge. Emerging literature reveals phenotypical and functional differences between peripheral blood and tissue neutrophils (12), but it is entirely unknown whether age-related changes in blood neutrophils mimic those in genital tissues.

Here we investigated how blood neutrophils and neutrophils from the female genital tract respond to HIV stimulation as women age. We focused on understanding how neutrophils recognize HIV, the molecular events that trigger the release of NETs, and how these mechanisms are modified as women age.

## Materials and methods

### Study subjects

This study was approved by the Committee for the Protection of Human Subjects (CPHS) and by the Health Sciences Institutional Review Board at Tufts University.

Genital tract tissue samples were obtained after surgery from HIV-negative women undergoing hysterectomies at Tufts Medical Center (Boston, MA, USA). Surgery was performed to treat benign conditions including fibroids, prolapse and menorrhagia. Trained pathologists selected tissue samples from ectocervix (ECX), endocervix (END), and endometrium (EM), free of pathological lesions and distant from the sites of pathology. Women were classified in this study as premenopausal (n=33; 24-55 years old; age (average)=42) and postmenopausal (n=9; 49-77 years old; age (average)=62). Menopausal status was self-identified.

Human blood samples were obtained from healthy female donors through Research Blood Components, LLC (Watertown, MA, USA). Women signed an Institutional Review Board (IRB) approved consent form where they gave permission to Research Blood Components, LLC to collect and sell their blood for research purposes. The only information provided about the donors was their age and menopausal status. Women were classified in this study as premenopausal women (n=42; 18-51 years old; age (average)=34) and as postmenopausal women (n=29; 48-72 years old; age (average)=60).

### Tissue processing and neutrophil purification

Tissues were processed to obtain a stromal cell suspension as described previously (16, 37, 39–41), with modifications. Enzymatic digestion was performed in gentleMACS™ Dissociator (Miltenyi Biotec, Auburn, CA, USA) using Tumor Dissociation Kit, human (Miltenyi Biotec) and 0.01% DNAse (Worthington Biochemical, Lakewood, NJ, USA). After filtering through a 100 μm, 70 μm, and 30 μm filters (MACS^®^ SmartStrainers; Miltenyi Biotec) to separate epithelial cells from stromal cells, cell suspensions underwent red blood cell removal (CD235a (Glycophorin A) MicroBeads, human; (Miltenyi Biotec)). Mixed cell suspensions were used for NET release experiments as indicated or underwent further processing to purify neutrophils. To isolate genital neutrophils, mixed cell suspensions were incubated first with CD66b Antibody, anti-human, PE, REAfinity™ (Miltenyi Biotec) followed by anti-PE MicroBeads UltraPure (Miltenyi Biotec) to be magnetically selected following the manufacturer’s instructions (Miltenyi Biotec) as described (37, 42).

### Neutrophil isolation from human peripheral blood

Venous blood from healthy women was collected into 10 mL EDTA tubes. Neutrophil isolation was performed as described (37, 38) by positive selection using CD15 MicroBeads (Miltenyi Biotec) and a whole blood column kit following the manufacturer’s instructions (Miltenyi Biotec).

### Inhibitors

Human purified neutrophils and mixed cell suspensions from genital tissues were resuspended in Hanks’ Balanced Salt Solution (HBSS) culture medium (Gibco; Waltham, MA, USA), plated in a 96-well plate (Corning Inc.; Corning, NY, USA) and pre-incubated at room temperature with the following inhibitors: TLR8 inhibitor at 5 µM for 1 h (CU-CPT9a; InvivoGen, San Diego, CA, USA), TBK1/IKKϵ inhibitor at 100 nM for 1 h (BX795, InvivoGen), TLR9 inhibitor at 150 nM for 30 min (ODN TTAGGG (A151), InvivoGen), TLR7 inhibitor at 150 nM for 30 min (ODN 2088 Control (ODN 20958), Miltenyi Biotec), TLR7/9 inhibitor at 150 nM for 30 min (Dual iODN; EnzoLifeSciences, Farmingdale, NY, USA), Dynamin inhibitor (Dynasore; Abcam, Waltham, MA, USA) at 200 µM for 20 min. Every inhibitor had its own control condition matching the media used to resuspend the inhibitors: DMSO, buffer solutions supplied with inhibitors or HBSS.

### NET induction and quantification with time-lapse microscopy

Following specific treatments, purified neutrophils from blood or genital tissues and mixed cell suspension were stimulated with 5000 GFP-labeled HIV-viral like particles (HIV-VLPs) per cell or calcium ionophore at 25 µM (A23187; Sigma-Aldrich, Saint Louis, MO, USA).

Extracellular DNA was quantified to determine the NET-HIV area with the IncuCyte software as described (37, 38). Cytotox red reagent at 250 nM (Essen Bioscience; Ann Arbor, MI, USA) was used to label DNA as described and annexin V dye at 1:200 dilution (IncuCyte^®^ Annexin V Dye for Apoptosis; Sartorius, Bohemia, NY, USA) was used to detect annexin V+ NETs. Images were collected every 3–5 min at 37°C using a 10x objective with the IncuCyte S3 (Sartorius) and NETs quantified as described (37, 38). Briefly, a mask was applied to determine colocalization of extracellular red fluorescent signal (DNA) and green fluorescent signal (HIV-VLPs). Intracellular signal within round small objects was excluded from the NET analysis. Because images were taken with a 10x magnification objective, single individual HIV-VLPs were undetectable. HIV-VLP fluorescent signal became detectable when multiple particles accumulated in NETs (37).

Cell membrane steadiness was assessed with Cytotox red reagent (Essen Bioscience). Positive cells were quantified using a mask that specifically selects round and small objects (cells).

### Calcium visualization and quantification with time-lapse microscopy

Rhod-3 AM (Rhod-3 Calcium Imaging Kit; Thermo Fisher, Waltham, MA, USA) was used to stain cytosolic calcium in red following manufacturer’s instructions. As an internal control of the positive signal, we used a calcium chelator (BAPTA, AM; Thermo Fisher) at 10 µM for 30 min at room temperature. To quantify the intracellular calcium area, we applied a mask to select intracellular red staining.

### ROS visualization and quantification with time-lapse microscopy

CellROX (CellROX™ Deep Red Reagent, Thermo Fisher) was used to stain in red cellular oxidative stress by reacting with ROS. CellROX dye is non-fluorescent when it is reduced but exhibits fluorescence once it is oxidated by ROS. As an internal control of the positive signal, we used a NADPH oxidase inhibitor (DPI; Sigma-Aldrich) at 10 µM for 30 min at room temperature. To quantify the intracellular ROS area, we applied a mask to select intracellular red staining.

### Generation of GFP-Labeled VLPs

Neutrophils were stimulated with CCR5-tropic GFP-labeled viral-like particles (VLPs) as described before (37, 38). GFP-labeled VLPs were generated using a modified pNL43 provirus-based plasmid for expression of GFP as described previously (43). The enhanced GFP (EGFP) coding sequence was expressed in the frame at the 3’end of the *gag*, replacing the protease and most of the reverse transcriptase coding region. The Ψ-signal on the RNA and the complete *gag* open reading frame (ORF) remained intact. To obtain CCR5 tropic strains (R5 strains), a plasmid with an inactivated Env ORF, resulting in no expression of functional Env protein (referred to as pNL4GagGSGFPDelta-env/K806), was derived from K795 for pseudotyping and then complemented with pBaL.26 Env expression plasmid (NIH AIDS Reagent program, catalog number 11,446, contributed by Dr. John Mascola) (44), generating VLPs with HIV-BaL envelope proteins (R5). For some experiments, VLP lacking envelope proteins (Delta-env) were used as indicated. Non-infectious, EGFP-labelled VLPs were produced by transfection, concentrated by ultracentrifugation, and enumerated essentially as described (43).

### Confocal microscopy

Cells were resuspended in HBSS and plated on coverslips pre-coated with poly-D-lysine (Gibco) for 20 min at room temperature. GFP-labeled HIV-VLPs (5,000-10,000 VLP/cell) were used to stimulate NET release for 5 min, 15 min, 30 min, 1h or 2 h at room temperature and then fixed with 4% paraformaldehyde. Unstimulated neutrophils incubated in HBSS were used as controls. Cells were stained for 2 h at room temperature with AlexaFluor 647-H3 (1B1-B2) (BioLegend) AlexaFluor 647-Rab5 (D-11) (Santa Cruz Biotechnology, Dallas, TX, USA), AlexaFluor 647-Lactoferrin (LF5-1D2) (Novusbio, Englewood, CO, USA), and/or PE-Annexin V (BioLegend, San Diego, CA, USA). Cells were carefully washed with PBS, and samples were mounted in Pro-Long Diamond anti-fade mounting with DAPI (Thermo Fisher) as described (37). Samples were imaged using a Leica SP8 confocal microscope in combination with Leica LAS X software.

### Flow cytometry

To determine neutrophil abundance in the FGT, mixed cell suspensions from genital tissues were stained for surface markers with combinations of the following antibodies: CD45-vioblue450, CD11b-PE (Tonbo, San Diego, CA), CD3-VioGreen, CD15-FITC, CD66b-APC (Miltenyi biotec), CD11c-PerCp-Cy5.5, CD3-APC (Biolegend), CD14-e780, (eBiosciences, San Diego, CA). Dead cells were excluded with 7AAD (Southern Biotech, Birmingham, AL, USA) or zombie dye yellow staining (Biolegend). Analysis was performed on 8-color MACSQuant 10 (Miltenyi biotec) or Gallios (Beckman Coulter, Indianapolis, IN, USA) flow cytometers and data analyzed with FlowJo software (Tree Star, Inc. Ashland, OR, USA). Expression of surface markers is shown as percentage of positive cells. Fluorescence minus one (FMO) strategy was used to establish appropriate gates. Cells were gated as described before (37).

To determine HIV receptor expression on neutrophils, purified blood neutrophils and mixed cell suspension from genital tissues were stained with the following anti-human antibodies: CD45-BUV395 (BD Biosciences), CD66b-Pacific blue (BioLegend), CD15-FITC (BioLegend), CD4-BUV805 (BD Biosciences), CXCR4-BV785 (BioLegend) and CCR5-PE-Cy5 (clone: 2D7/CCR5) (BD Biosciences). Cell viability was assessed with a live/dead fixable blue dead cell stain kit (Thermo Fisher) used for 10 min in a 1:200 dilution. Cells were incubated with FcR Blocking Reagent (Miltenyi biotec) for 10 min (5µl/1M cells) to block Fc receptors. Analysis was performed on Aurora cytometer (Cytek Biosciences; Fremont, CA, USA) and assessed with OMIQ (www.omiq.ai).

### ELISA

Invitrogen™ Estradiol Human ELISA Kit (Thermo Fisher) was used to determine estradiol levels in plasma from blood donors following manufacturer’s instructions. Plasma samples were obtained from blood (1 ml) after centrifugation at 500g/15min/RT.

### Quantitative polymerase chain reaction

Human blood neutrophils were processed using RNeasy Plus Mini Kit (Qiagen; Hilden, Germany) to extract and purify RNA. Next, high-quality RNA was obtained by removing contaminants using RNeasy cleanup kit (Qiagen). *TLR7*, *TLR8*, *TLR9*, and *RPL13a* (as internal control gene) genes were amplified using *Power*SYBR™ Green PCR Master Mix (Thermo Fisher) and the following primer pairs: TLR7, hFw 5’tttcccagagcatacagcttag3’ and hRv 5’gcctctgatgggacaagtaaa3’; TLR8, hFw 5’gctacaggtctctttccacatc3’ and hRv 5’gtggtagcgcagctcattta3’; TLR9, hFw 5’gctagacctgtcccacaataag3’ and hRv 5’aaagggctggctgttgtag3’; RPL13a, hFw 5’gccctacgacaagaaaaagcg3’ and hRv 5’tacttccagccaacctcgtga3’. Samples were run on the QuantStudio 6 Flex System. All kits were used according to manufacturer’s instructions.

### Statistical analysis

Data analysis was performed using the GraphPad Prism 9 software. Data are represented as median ± interquartile range. One and two-sided p-value ≤ 0.05 were considered statistically significant as indicated. Non-parametric Mann–Whitney U test or Wilcoxon’s matched pair test was used for comparison of two groups. One sample t test was used to determine whether an unknown population is different from a hypothetical value, assuming sampling from a Gaussian distribution. Non-parametric Spearman’s correlation was used for correlation analysis.

## Results

### NET release in response to HIV is delayed with aging after menopause.

Previously, we and others have demonstrated that neutrophils from blood and genital tissues release NETs in response to HIV (36–38). Here we wanted to investigate the impact of aging on HIV-induced NET release in blood and genital tissues. To do this, we stimulated blood and genital neutrophils from women (18-77 years of age) with GFP-labeled HIV viral like particles (VLPs) expressing CCR5-tropic envelope proteins (HIV-BaL) to visualize and quantify NET-release using time-lapse microscopy as described previously (37, 38).

**Figure 1:**
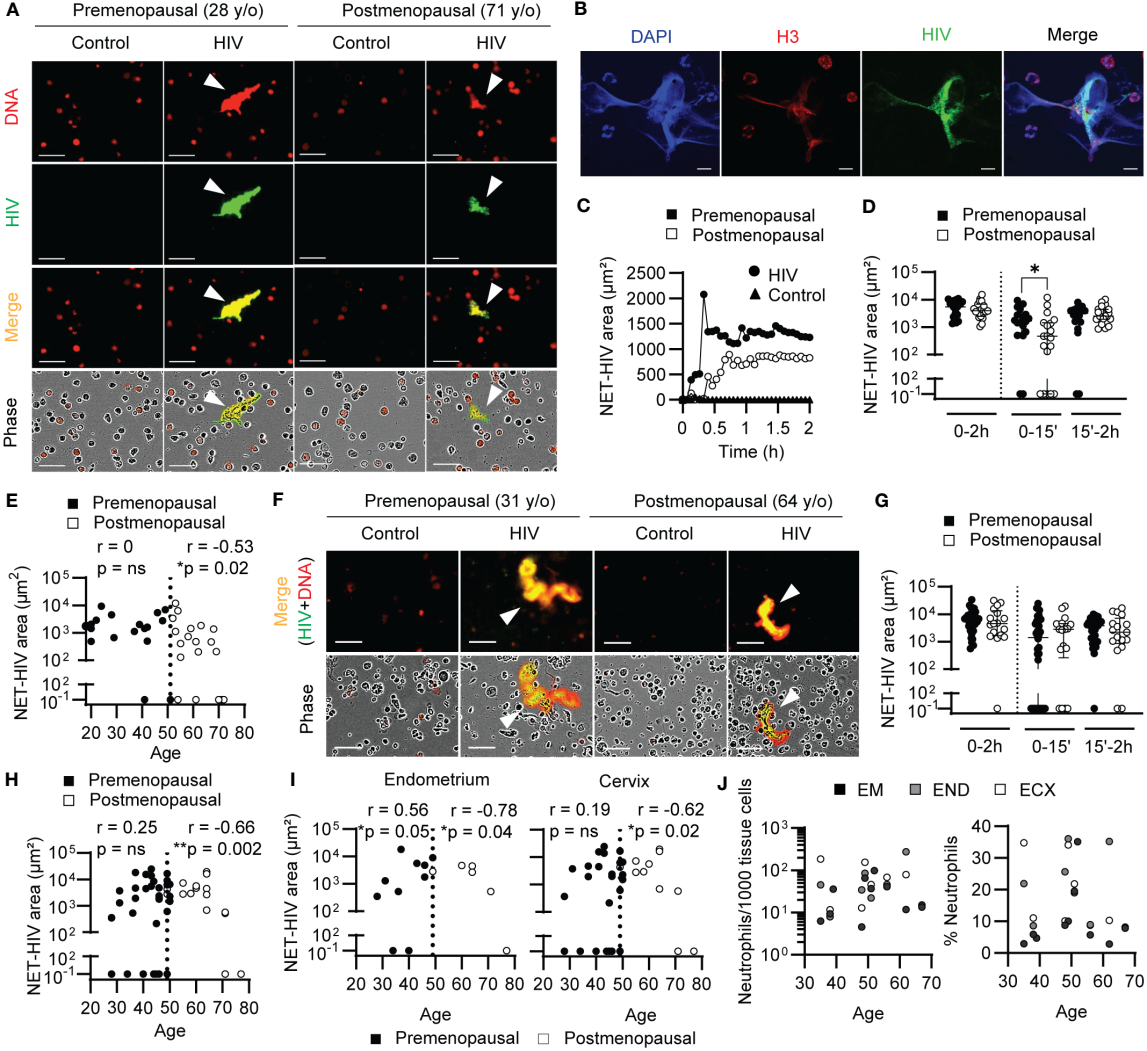
Aging delays NET release in response to HIV. **(A)** Representative NET release images by blood neutrophils after HIV stimulation (2h). Arrows indicate NETs. Scale bar: 50μm. **(B)** Representative confocal images of HIV-NETs after 1h stimulation. Scale bar: 10 μm. **(C)** Representative quantification of NET release over time by premenopausal (black) and postmenopausal women (white) after HIV stimulation (circles). Unstimulated neutrophils (triangles) overlap at the bottom. **(D)** Comparison of NET release by blood neutrophils from premenopausal (n=18) and postmenopausal women (n=19) at total (0-2h), early (0-15min), late (15min-2h) times. Mann-Whitney test. Data represent median with interquartile range. **(E)** Correlation of early NET release (15min) and women’s age (premenopausal=18; postmenopausal=19) in blood. Spearman r correlation. Vertical dotted line indicates menopause. **(F)** Representative NET release images by genital neutrophils after HIV stimulation (2h). Scale bar: 50μm. **(G)** Comparison of NET release by genital neutrophils from premenopausal (n=34) and postmenopausal women (n=17) at total (0-2h), early (0-15min) and late (15min-2h) times. Mann-Whitney test. Data represent median with interquartile range. **(H)** Correlation of early NET release (15min) and women’s age (premenopausal=34; postmenopausal=17) in genital tissues and **(I)** separated by anatomical compartment: endometrium and uterine cervix (endocervix + ectocervix). Spearman r correlation. Vertical dotted line indicates menopause. **(J)** Correlation between women’s age and number of neutrophils (left graph) or neutrophil percentage within immune cells (CD45+) (right graph) in endometrium (n=11), endocervix (n=7) and ectocervix (n=7). *p<0.05; **p<0.01.

Consistent with our previous findings (37, 38), we detected NET release after stimulation of blood neutrophils with GFP-labeled HIV-VLPs compared to control condition (**Figure 1A**). To further confirm the presence of HIV-NET complexes by an alternative method, HIV-NETs were visualized using confocal microscopy. Neutrophils were plated on glass slides and stimulated with GFP-labeled HIV-VLPs to induce the release of NETs, followed by staining of DNA and histone 3 (H3), a NET-associated protein (20). As shown in **Figure 1B**, we observed colocalization of HIV, extracellular DNA and H3, indicating HIV entrapment in NETs. Interestingly, a reduction in NET formation was visualized in postmenopausal when compared to premenopausal women (**Figure 1A**). Kinetic quantification of NET release by blood neutrophils overtime from premenopausal women identified a rapid initial peak of NET release (early NET release; 0-15min), followed by sustained release over the next 2h (late NET release; 15min-2h) (**Figure 1C**; black circles). In contrast, the kinetics of NET release in postmenopausal women revealed the absence of an initial peak of NET release following HIV stimulation which progressively increased over time (**Figure 1C**; white circles). Quantification of total NET release in multiple women (n=37) at 2h after HIV stimulation did not detect any differences between pre and postmenopausal women (**Figure 1D**; 0-2h); however, we detected significantly lower release of NETs in postmenopausal compared to premenopausal women at early time points (**Figure 1D**; 0-15min, p=0.02), with no significant differences at later time-points after HIV stimulation (**Figure 1D**; 15-2h). These results suggest a delay in the response of blood neutrophils to the virus in postmenopausal women compared to premenopausal women. Recognizing that menopause is a critical point in the aging process for women that marks the end of sex hormones cycles (18), we next investigated whether the observed effects in early NET release were influenced by aging following menopause. As seen in **Figure 1E**, during the premenopausal years, early NET release was maintained as women aged (**Figure 1E**; black circles), but significantly declined with aging following menopause (**Figure 1E**; white circles, r=-0.53, p=0.02).

To determine if this decline in NET release was directly influenced by sex hormones, we measured estrogen (E2) levels in plasma samples from 24 premenopausal and 25 postmenopausal women, including 28 samples used for NET release experiments (**Figure S1**). As expected, premenopausal women showed significantly higher levels of E2 in plasma compared to postmenopausal women (**Figure S1A**). Next, we analyzed pre and postmenopausal women groups independently to identify correlations between E2 levels and age in our cohort. For premenopausal women, we observed no significant correlation between the age of premenopausal women and their E2 levels (**Figure S1B**). This is consistent with the known high variability between women, and between cycles for the same woman as reported previously (11, 45–48). Additionally, we noticed a broad variability in the E2 levels in the 40-50-year-old range, with lower levels after 45. In the postmenopausal group, there was also no significant correlation between age and plasma E2 levels, which remained low and constant (**Figure S1B**). Importantly, when we correlated E2 plasma levels from every donor with HIV-NET release by purified blood neutrophils, there was no significant correlation (**Figure S1C**), indicating that the decrease in E2 levels in postmenopausal women is not directly influencing the release of HIV-induced NETs.

Next, we investigated NET release by genital neutrophils (**Figure 1F**). Similar to blood neutrophils, we visualized a general reduction in NET release between younger and older women. However, in contrast to our results with blood neutrophils, when NET release from multiple women was quantified, we did not detect a significant difference in NET release between pre and postmenopausal women, whether total, early or late NET release (**Figure 1G**). However, when NET release was correlated with age, genital neutrophils from postmenopausal women significantly decreased the early NET release progressively as women aged (**Figure 1H**, white circles; r=-0.66, p=0.002). To determine if aging differentially affected neutrophils from different anatomical parts of the FGT, we stratified the results in **Figure 1H** according to FGT site (endometrium and cervix) in pre and postmenopausal women. As seen in **Figure 1I**, endometrium and cervix neutrophils displayed similar correlation between early NET release and aging in women (**Figure 1I**). Unexpectedly, NET release by endometrial neutrophils from premenopausal women significantly increased with the women’s age until they reached the perimenopausal years (**Figure 1I**; left graph), and the same trend was observed in the cervix, likely contributing to the lack of differences detected between pre and postmenopausal women when considered as a whole (**Figure 1G**). To determine if the observed correlation between reduced NET release and increasing age was associated with a decrease in neutrophil presence in the FGT, we measured the number of neutrophils and percentage of neutrophils in genital tissues by flow cytometry as described before (37). As seen in **Figure 1J**, neutrophil presence and percentage remained constant with increasing age in the different anatomical sites of the FGT.

Overall, these results demonstrate that aging impacts the ability of neutrophils from blood and the FGT to release NETs in response to HIV.

### TLR8 and TLR7 mediate early and late HIV-induced NET-release in a temporal sequential manner

Since neutrophils from postmenopausal women demonstrated a defect in the early NET release response following HIV stimulation, we next investigated HIV recognition by neutrophils to determine if this mechanism was impaired with aging.

HIV ssRNA is recognized by TLR7/8 (49) and stimulation of these TLRs results in neutrophil activation and NET release (36, 37, 50). Therefore, we investigated the role of TLR7/8 in HIV recognition by blood and genital neutrophils and their involvement in early NET release.

**Figure 2:**
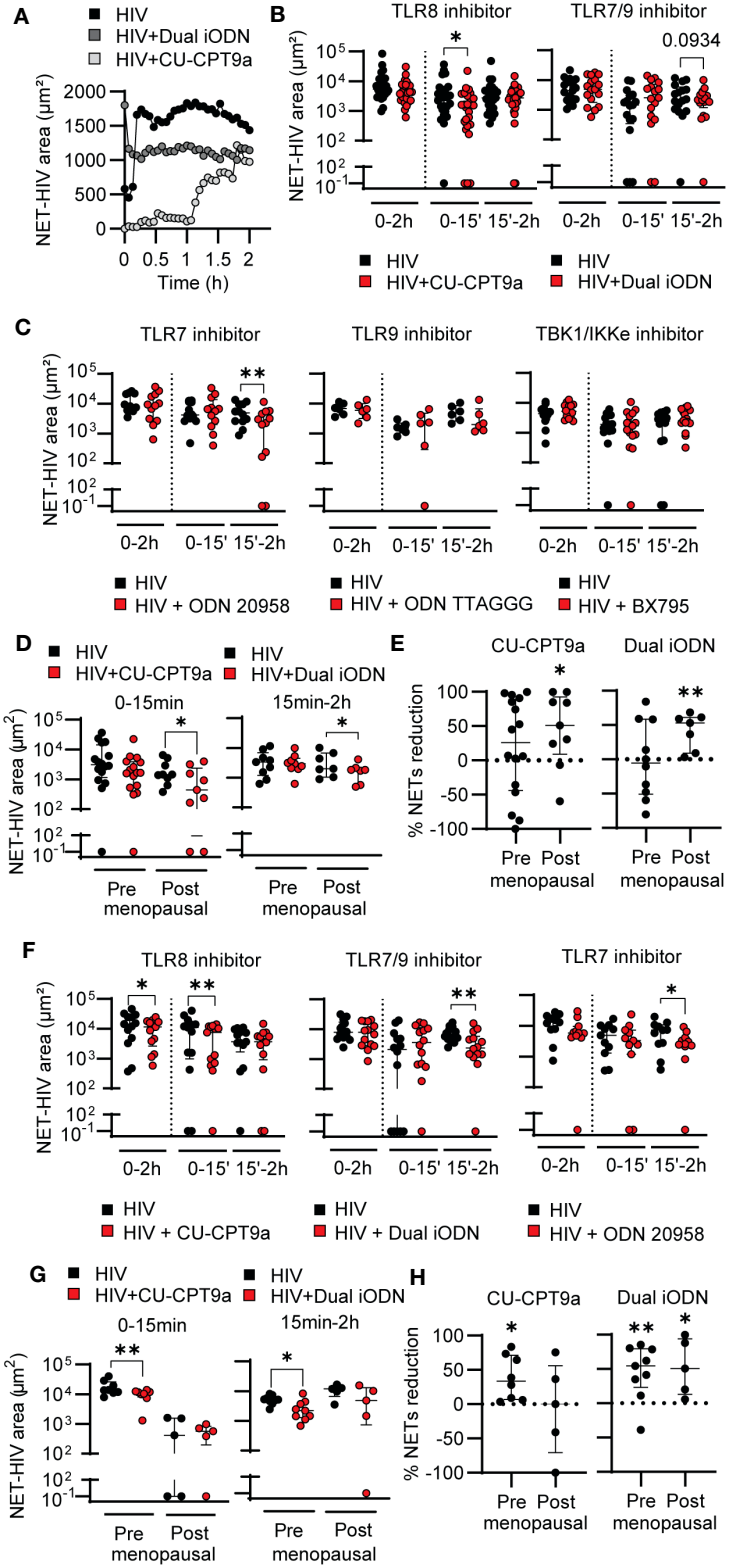
TLR8 and TLR7 mediate HIV-induced NET-release in a temporal sequential manner. **(A)** Representative quantification of HIV-induced NET release over time in HIV only condition (black), dual iODN (dark gray), or CU-CPT9a (light gray) treatment in blood neutrophils (CU-CUPT9a: n=25; Dual iODN: n=17). **(B, C)** NET release in HIV alone condition (black) or after TLR or RLR inhibition (red) in blood neutrophils. Wilcoxon test. 1 outlier removed from ODN 20958 condition by Grubbs’s test (Alpha = 0.05). **(D)** NET release by blood neutrophils from premenopausal and postmenopausal women after TLR8 (left graph) and TLR7/9 (right) inhibition. Wilcoxon test. **(E)** Normalized reduction in NET release after TLR8 (left graph) or TLR7/9 (right graph) inhibition with respect to HIV only control condition in blood neutrophils from pre and postmenopausal women. One sample t test **(F)** NET release by genital neutrophils in response to HIV stimulation alone (black) and following treatment with TLR inhibitors (red) (CU-CUPT9a: n=13; Dual iODN: n=14; ODN 20958: n=11). Premenopausal women’s age (average): 44; postmenopausal women´s age (average): 60. Wilcoxon test. 1 outlier removed from ODN 20958 condition by Grubbs’s test (Alpha = 0.05). **(G)** NET release in response to HIV by genital neutrophils from premenopausal and postmenopausal women following treatment with TLR inhibitors. Wilcoxon test. **(H)** Normalized reduction in NET release after TLR8 (left graph) or TLR7/9 (right graph) inhibition with respect to HIV only condition in genital neutrophils from pre and postmenopausal women. One sample t test; horizontal lines represent median with interquartile range. *p<0.05, **p<0.01.

To do this, we incubated blood neutrophils with specific TLR inhibitors prior to HIV stimulation and quantified NET formation overtime. In blood neutrophils, as shown in a representative example, TLR8 inhibition (CU-CPT9a) delayed the kinetics of NET release compared to HIV alone (**Figure 2A**; **Supplementary Movie 1**), while inhibition of TLR7 and TLR9 (TLR7/9: Dual iODN) impacted NET release at later time points (**Figure 2A**; **Supplementary Movie 2**). Quantification of HIV-NET release by blood neutrophils from multiple women after blocking TLR8 with CU-CPT9a demonstrated significant reduction (45% median reduction; p=0.03) at early time points (0-15 min), with no significant changes at later time points (15min-2h) (**Figure 2B**, left panel). In contrast, TLR7/9 inhibition with Dual iODN trended to reduce the release of NETs at late time points (**Figure 2B**, right panel). Because Dual iODN inhibits TLR7 and TLR9, to determine whether the observed effect was due to TLR7 or TLR9 signaling, we used specific TLR7 (ODN 20958) or TLR9 (ODN TTAGGG) inhibitors and observed a significant reduction in late NET release after TLR7 inhibition (**Figure 2C**; 59% median reduction; p=0.007), but no effect with TLR9 inhibition (**Figure 2C**). We also tested the contribution of additional pattern recognition receptors (PRRs) able to detect viral RNA by inhibiting TANK-binding kinase 1 (TBK1) and I-kappa-B kinase epsilon (IKKϵ) before HIV stimulation, two IκB kinase homologs which are indispensable for the signaling downstream of RIG-I-like receptors (RLRs) (51). However, we did not detect significant differences in the release of NETs (**Figure 2C**, right panel). Our findings indicate that blood neutrophil recognition of HIV through TLR8 mediates early NET release, while TLR7 preferentially mediates late NET release.

To evaluate the effects of menopause and aging on HIV recognition and NET release by blood neutrophils, we stratified the data in **Figure 2B** into pre and postmenopausal women. We hypothesized that HIV recognition would be impaired in postmenopausal women as a result of reduced TLR signaling and this would be responsible for the delay in HIV-induced NET release (Figure1). Unexpectedly, inhibition of TLR8 and TLR7/9 significantly reduced NET release in the postmenopausal group, with no significant effect in premenopausal women (**Figure 2D**). Furthermore, normalization of HIV-induced NET release in the presence of the TLR inhibitor versus in the presence of HIV only (control condition) revealed that the percentage of NET reduction was enhanced in postmenopausal compared to premenopausal women (**Figure 2E**). We next assessed TLR7, TLR8 and TLR9 expression to determine if the observed differences in endosomal TLR signaling between pre and postmenopausal women were due to age-dependent changes in TLR expression. However, we did not detect differences between pre and postmenopausal women, and TLR8 was expressed at higher levels than the other TLRs (**Figure S2**), consistent with prior publications (52).

Overall, these data indicate that blood neutrophil recognition of HIV through TLR8 mediates early NET release, while TLR7 preferentially mediates late NET release. Further, TLR8 and TLR7 signaling preferentially mediates HIV-induced NET-release after menopause, suggesting that premenopausal women possess alternative mechanisms responsible for triggering NET release in response to HIV stimulation.

Next, we analyzed genital neutrophils. TLR8 inhibition significantly reduced early NET release (28.2% median reduction; p=0.005) (**Figure 2F**, left panel) but to a lesser extent than in blood neutrophils (**Figure 2B**, left panel). TLR7/9 and TLR7 inhibition reduced late NET release (48% median reduction; p=0.002) (**Figure 2F**). In contrast to blood neutrophils, when pre and postmenopausal women were analyzed separately, genital neutrophils from premenopausal women reduced the release of NETs in response to HIV when TLR8 was inhibited, but no effect of inhibition was detected in postmenopausal women (**Figure 2G**, left panel), potentially due to the already low response to HIV alone. Similarly, NET reduction was detected after TLR7/9 inhibition only in premenopausal women (**Figure 2G**, right panel). Normalization of HIV-induced NET release in the presence of the TLR inhibitor respect to HIV only demonstrated a reduction in the effect of TLR8 inhibition as women aged, while TLR7 inhibition remained equally effective in pre and postmenopausal women treated with Dual iODN (**Figure 2H**). These results suggest that HIV-induced NET release in genital neutrophils is preferentially induced through TLR8 and TLR7 in a sequential manner, and that TLR8 signaling mechanisms are impaired as women age.

Overall, these data demonstrate that HIV triggers NET release in blood and genital neutrophils through differential mechanisms and that aging specifically impairs TLR8 signaling in the FGT.

### HIV stimulation specifically induces rapid cytosolic calcium upregulation in blood neutrophils from younger women

The lack of NET reduction after inhibition of TLR8 and TLR7 signaling in HIV-stimulated blood neutrophils from younger women suggested the presence of alternative NET-triggering mechanisms. Furthermore, although TLR8 and TLR7 inhibition resulted in a significant reduction of early and late NET release in blood neutrophils from older women, NET formation was not completely abrogated, suggesting the existence of additional activation mechanisms. Thus, we explored the potential role of intracellular calcium, which has been described as a rapid mediator of NET release in response to bacteria (33–35).

**Figure 3:**
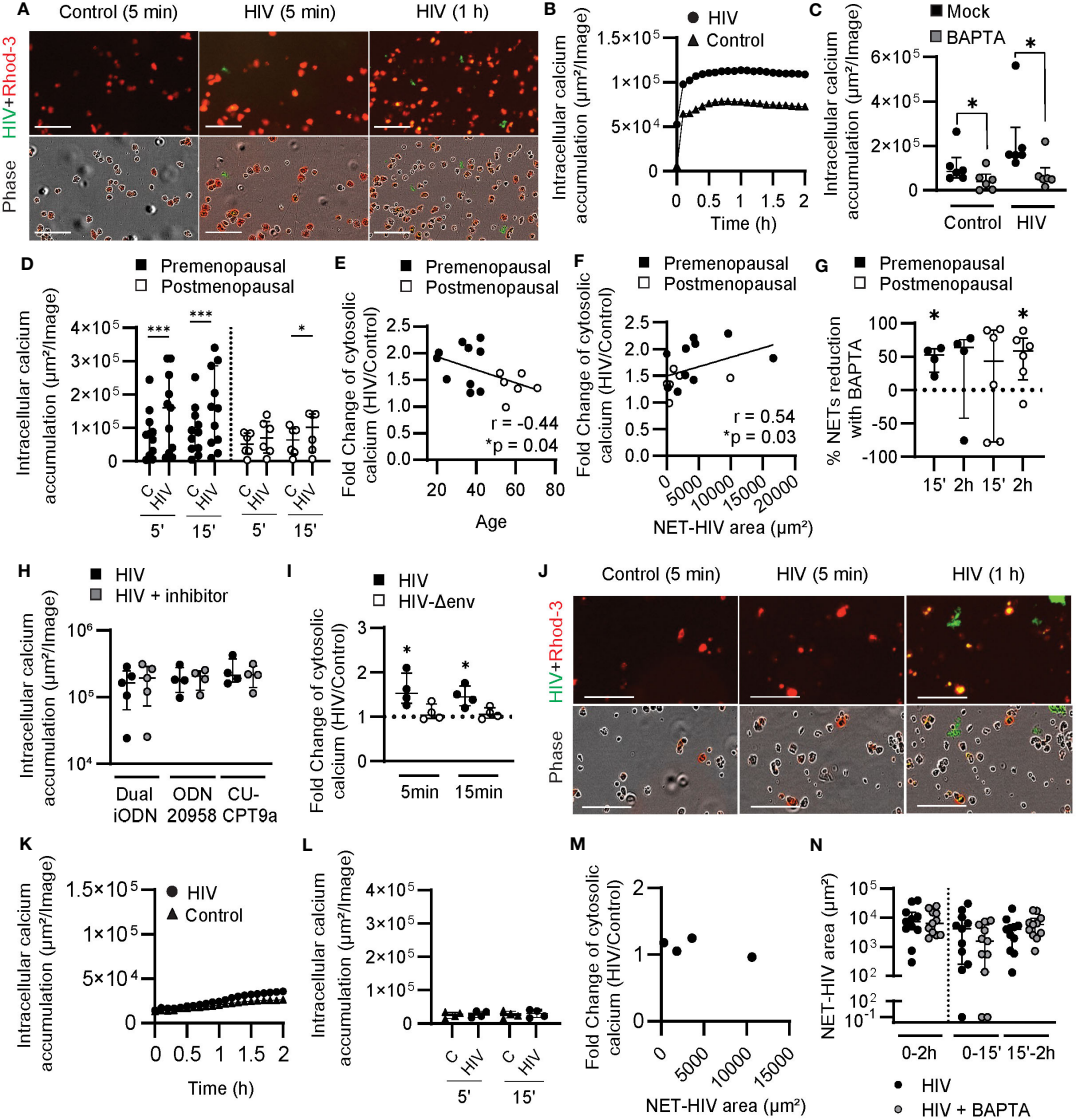
Induction of cytosolic calcium by HIV stimulation in blood neutrophils from younger women. **(A)** Representative images of intracellular calcium increase (Rhod-3 AM) in blood neutrophils after HIV stimulation (HIV-GFP, green). **(B)** Representative quantification of intracellular calcium in blood neutrophils after HIV stimulation. **(C)** Comparison of intracellular calcium levels in the absence (black) or presence (gray) of BAPTA in resting blood neutrophils and after HIV stimulation (5 min). Wilcoxon test. **(D)** Comparison of intracellular calcium accumulation in blood neutrophils from pre (n=11; black) and postmenopausal women (n=6; white) after 5 and 15 min of HIV stimulation. Wilcoxon test. **(E)** Correlation between age and intracellular calcium increase after HIV stimulation at 5 min. Spearman r correlation (one-tailed). **(F)** Correlation between early NET release by blood neutrophils and intracellular calcium increase after HIV stimulation at 5 min. Spearman r correlation (two-tailed). **(G)** Percentage of HIV-induced NETs reduction in the presence of BAPTA in blood neutrophils from premenopausal (black) and postmenopausal (white) women. One sample t test. **(H)** Lack of effect of TLR inhibition in intracellular calcium levels in blood neutrophils stimulated with HIV. **(I)** Intracellular calcium increase in blood neutrophils stimulated with HIV with envelope (black) or HIV without envelope (white). One sample t test. **(J)** Representative images of calcium staining (Rhod-3 AM) in genital neutrophils after HIV stimulation (HIV-GFP, green). **(K)** Representative quantification of intracellular calcium in genital neutrophils after HIV stimulation. **(L)** Intracellular calcium accumulation in genital neutrophils after HIV stimulation (circles) or control condition (triangles). **(M)** Lack of correlation between early NET release by genital neutrophils and intracellular calcium after HIV stimulation. **(N)** NET release by genital neutrophils after HIV stimulation (black) or HIV stimulation plus BAPTA (gray). Horizontal lines represent median with interquartile range. *p<0.05, ***p<0.001. Scale bar: 100µm.

Blood neutrophils were pre-incubated with red fluorescent Rhod-3 AM dye to measure intracellular calcium levels with and without HIV stimulation. As seen in **Figure 3A** and **Supplementary Movie 3**, intracellular calcium fluorescence signal was increased after HIV stimulation compared to control conditions. Kinetic quantification showed a very rapid increase in intracellular calcium within 5 min of HIV treatment, which peaked approximately 20 min after stimulation and was then maintained (**Figure 3B**). As an additional control to confirm that HIV was responsible for the intracellular calcium increase detected, we incubated blood neutrophils with BAPTA, an intracellular calcium chelator, prior to HIV stimulation. As seen in **Figure 3C**, BAPTA treatment significantly reduced intracellular calcium levels in the absence of HIV stimulation and abrogated the intracellular calcium response to HIV.

Next, we compared blood neutrophils from pre and postmenopausal women. Neutrophils from premenopausal women significantly upregulated intracellular calcium 5 min after stimulation and this difference remained significant at 15 min (**Figure 3D**). In contrast, neutrophils from postmenopausal women did not significantly upregulate intracellular calcium until 15 min following stimulation, with the overall amount of intracellular calcium lower in post compared to premenopausal women (**Figure 3D**). We then investigated whether intracellular calcium levels after HIV stimulation were affected by aging following menopause and detected a negative and significant correlation between the fold-change in intracellular calcium in response to HIV stimulation and women’s age (**Figure 3E**; r=-0.44; p=0.04).

To determine if intracellular calcium levels were associated with early NET release, we quantified NET release at 15 min and tested for correlation of NET-HIV area with the fold-change in intracellular calcium following HIV stimulation. As seen in **Figure 3F**, there was a positive and significant correlation between early NET release and intracellular calcium responses to HIV (r=0.54; p=0.03). This data suggests that intracellular calcium levels mediate early NET release in younger women. To test this, we quantified NET release in the presence of BAPTA in pre and postmenopausal women and detected a significant reduction in early NET release after HIV stimulation in premenopausal women (**Figure 3G**; 53% median reduction at 15 min), but a delayed effect in reduction of NET release in postmenopausal women (**Figure 3G**; 59% median reduction at 2h). These data demonstrate that early NET release in blood neutrophils from premenopausal women is triggered by intracellular calcium increase, but this response is delayed in postmenopausal women.

To determine if increased intracellular calcium levels in response to HIV stimulation were a result of HIV recognition by endosomal TLRs, cytosolic calcium levels were measured in blood neutrophils treated with endosomal TLR inhibitors prior to HIV stimulation. Interestingly, we did not observe any changes in intracellular calcium when TLR7/9 (Dual iODN), TLR7 alone (ODN 20958) or TLR8 (CU-CPT9a) were blocked prior to HIV stimulation (**Figure 3H**), indicating that calcium release was not triggered by endosomal viral recognition. We then explored whether the cytosolic calcium response was triggered by viral engagement of plasma membrane receptors by comparing HIV-VLPs with and without viral envelope (Env) proteins. As seen in **Figure 3I**, intracellular calcium was increased in response to HIV-VLPs with Env, and no changes were observed in the absence of Env proteins (Δenv), indicating that the intracellular calcium response was driven by the HIV Env, and not by the viral RNA genome.

Next, we investigated genital neutrophils. To evaluate calcium responses, we purified genital neutrophils as previously described (37) prior to HIV stimulation and quantification of intracellular calcium. As seen in **Figure 3J** and **Supplementary Movie 4**, low levels of intracellular calcium were detected in genital neutrophils following HIV stimulation. Kinetic quantification demonstrated the lack of an intracellular calcium response compared to unstimulated control condition (**Figure 3K**). Analysis of multiple donors (n=4) did not detect intracellular calcium increase after HIV stimulation (**Figure 3L**). Importantly, although HIV stimulation did not increase calcium levels in genital neutrophils, these neutrophils were able to release NETs (**Figure 3J**; green), but no correlation was found between NET release and intracellular calcium levels (**Figure 3M**). Furthermore, BAPTA did not reduce the NET release (**Figure 3N**), indicating that calcium is not a mediator for the release of NETs by genital neutrophils in response to HIV stimulation.

Overall, these findings indicate fundamentally different mechanisms for NET release between blood and genital neutrophils in response to HIV, with increase of intracellular calcium triggered only in blood neutrophils after recognition of the HIV envelope and in an age-dependent manner.

### HIV stimulation induces ROS production through differential mechanisms in blood and genital neutrophils

We next investigated the production of ROS, which has been implicated in NET release in response to HIV stimulation (36, 37), as an additional possible mediator of the observed effects.

**Figure 4:**
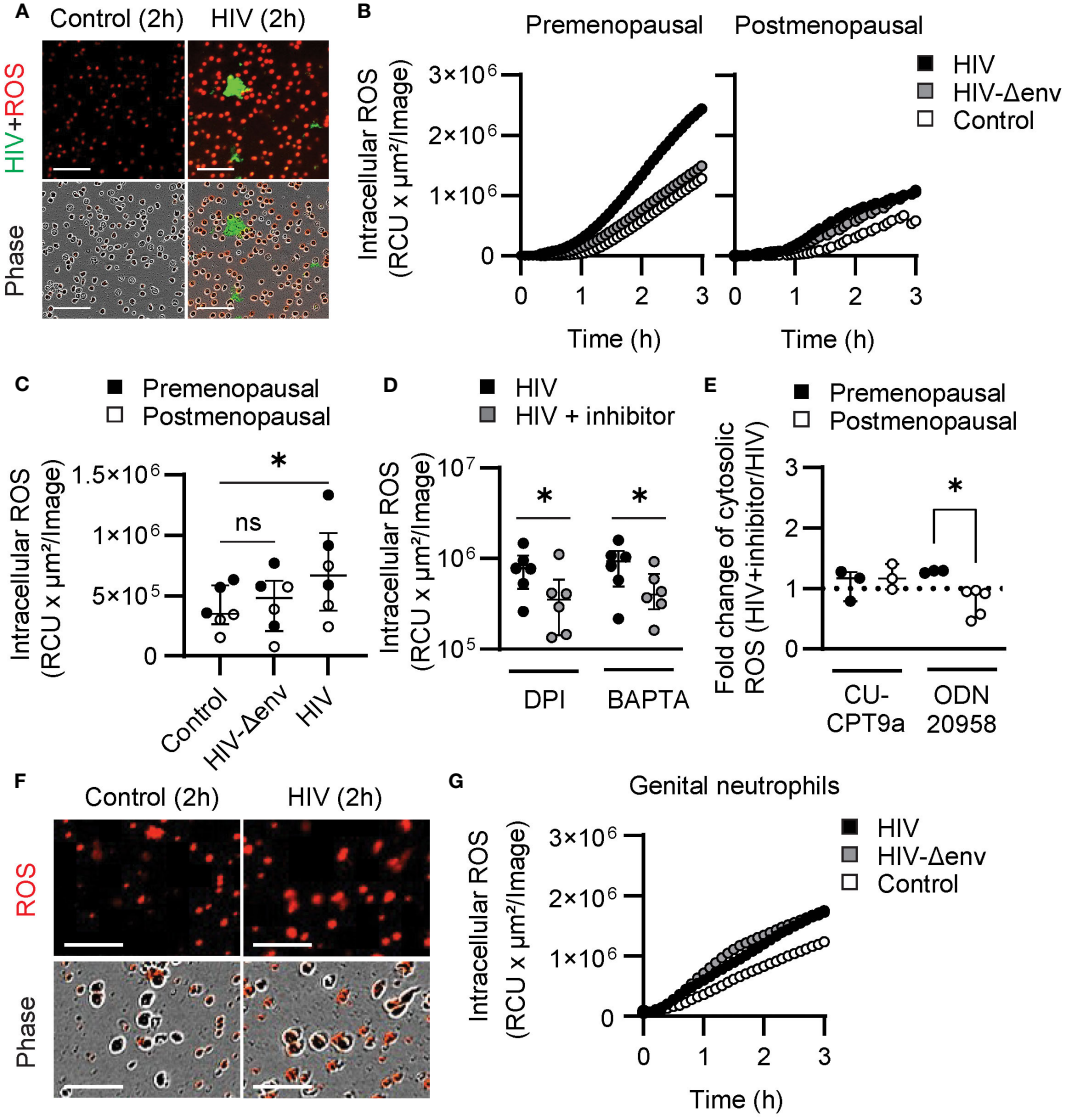
HIV stimulation induces ROS production through differential mechanisms in blood and genital neutrophils. **(A)** Representative images of blood neutrophils stained with CellROX dye (red) prior to stimulation with HIV-VLPs (HIV-GFP, green). Scale bar: 100µm. **(B)** Representative quantification of intracellular ROS production by blood neutrophils in control condition (white) or after stimulation with HIV (black), or HIV lacking envelope proteins (HIV-Δenv) (gray). **(C)** Intracellular ROS production by blood neutrophils from premenopausal (black) and postmenopausal (white) women after HIV or HIV-Δenv stimulation. Wilcoxon test. **(D)** Intracellular ROS production by blood neutrophils stimulated with HIV alone (black) or in the presence of NADPH oxidase inhibitors (DPI) or calcium chelators (BAPTA). Wilcoxon test. **(E)** Fold-change of intracellular ROS production after HIV stimulation in the presence of TLR inhibitors ((HIV+TLR inhibitor)/HIV) by blood neutrophils from premenopausal (black) and postmenopausal (white) women. Mann–Whitney U test. **(F)** Representative images of genital neutrophils stained with CellROX dye (red) after stimulation with HIV-VLPs (HIV-GFP, green). Scale bar: 50µm. **(G)** Representative quantification of intracellular ROS production by genital neutrophils in control condition (white) or after stimulation with HIV (black), or HIV lacking envelope proteins (HIV-Δenv) (gray). *p<0.05.

Blood neutrophils were incubated with CellROX Deep Red, a cell-permeant dye that becomes fluorescent when oxidized by ROS, to visualize and measure intracellular ROS production with and without HIV stimulation. As seen in **Figure 4A** and in **Supplementary Movie 5**, HIV stimulation enhanced intracellular ROS production in HIV-stimulated neutrophils compared to resting neutrophils. Next, we quantified intracellular ROS production in pre and postmenopausal women after stimulation with HIV-VLPs with and without Env. Quantification of intracellular ROS revealed increased production after 1 h of HIV stimulation, but only with HIV-VLPs containing Env glycoproteins (**Figures 4B, C**), and this response was impaired in postmenopausal women (**Figures 4B, C**). Since intracellular ROS production and calcium increase seemed to be dependent on engagement of viral Env proteins, we next investigated whether calcium increase could be triggering intracellular ROS production in response to HIV stimulation. Thus, we measured intracellular ROS in neutrophils that were pre-incubated with BAPTA, and we used DPI (a NADPH oxidase inhibitor) as a positive control to inhibit ROS production. Interestingly, we found that BAPTA significantly reduced intracellular ROS levels when neutrophils were stimulated with HIV, and this reduction was similar in magnitude to the reduction obtained after inhibition of NADPH oxidase using DPI (**Figure 4D**). This suggests that intracellular calcium increase triggers the production of ROS. Finally, to determine if HIV recognition through endosomal TLRs led to NADPH oxidase activation and ROS production, we measured intracellular ROS in blood neutrophils treated with TLR7 inhibitor (ODN20958) and TLR8 inhibitor (CU-CPT9a). Only TLR7 inhibition reduced ROS production in response to HIV stimulation in blood neutrophils from postmenopausal women, but no effect was detected in premenopausal women or in women of all ages after TLR8 blockade (**Figure 4E**). These results indicate that in blood neutrophils HIV-induced calcium increase triggers ROS production in a TLR-independent manner preferentially in premenopausal women, while in postmenopausal women ROS production is preferentially induced by HIV-induced signaling through TLR7.

Next, we investigated genital neutrophils. To evaluate ROS responses, we purified genital neutrophils as described (37) and we quantify intracellular ROS with and without HIV stimulation. As visualized in **Figure 4F**, genital neutrophils enhanced intracellular ROS production after HIV stimulation compared to resting neutrophils, and ROS production was initiated at 30 min (**Figure 4G**), earlier than the response detected for blood neutrophils (**Figure 4B**). Further, in contrast to blood neutrophils, ROS production was not dependent on viral envelope stimulation since no differences were found after stimulation with HIV-VLP with or without the envelope (**Figure 4G**), suggesting that ROS production was induced after endosomal TLR activation.

Overall, these findings further support differential signaling pathways after HIV stimulation in blood and tissue neutrophils, with blood neutrophils responding to HIV viral Env proteins with upregulation of calcium and ROS production in an age-dependent manner, and genital neutrophils responding with rapid ROS production in a calcium-independent and HIV-Env independent manner.

### HIV is internalized by a subset of neutrophils

In light of our results demonstrating that HIV can activate membrane receptors and also endosomal TLRs in neutrophils, we next investigated potential mechanisms for viral internalization.

**Figure 5:**
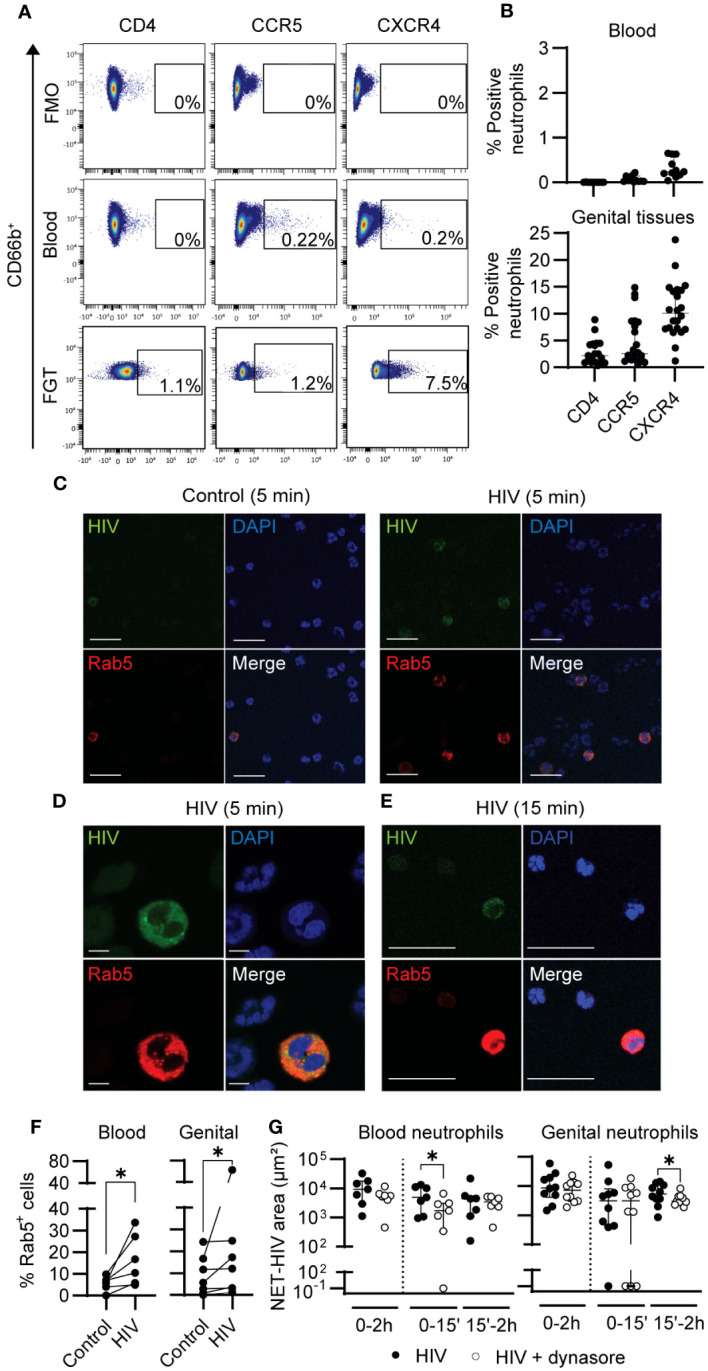
HIV is internalized by neutrophils. **(A)** Representative flow cytometry plots for CD4, CCR5 and CXCR4 expression on blood and genital neutrophils. **(B)** Percentage of expression of CD4, CXCR4, and CCR5 in blood neutrophils (top graph) and in genital neutrophils (bottom graph). **(C)** Representative images of confocal microscopy of Rab5 expression (red) in blood neutrophils before (control) or after 5 min of HIV stimulation. Scale bar: 25 µm. **(D)** Representative confocal image (zoom) of Rab5+ blood neutrophils after 5 min of HIV stimulation. Scale bar: 50 µm. **(E)** Representative images of Rab5+ genital neutrophils after 15 min of HIV stimulation using confocal microscopy. Scale bar: 25 µm. **(F)** Quantification of the percentage of Rab5+ cells in resting versus HIV-stimulated blood neutrophils (left graph) and genital mixed cell suspensions (right graph). Wilcoxon test. **(G)** NET release in response to HIV stimulation (black) and HIV stimulation in the presence of dynamin inhibitor (white) in blood neutrophils (left graph) and genital neutrophils (right graph). Wilcoxon test. Horizontal lines represent median with interquartile range. *p<0.05.

Neutrophils are not considered as HIV-target cells, although the presence of a subset of peripheral blood neutrophils that express CD4 has been reported in some individuals (53, 54). Thus, we first determined if blood and genital neutrophils express the HIV receptor CD4, and coreceptors CCR5 and CXCR4. As shown in a representative example in **Figure 5A**, and with multiple patients (blood: n=11; tissue samples: n=22) (**Figure 5B**), we did not detect CD4 expression in blood neutrophils, and CXCR4 and CCR5 expression were detected in less than 1% of cells. In contrast, in genital neutrophils (**Figures 5A, B**), HIV receptor (CD4) was detectable in a small percentage of cells (2.2%) and coreceptors (CCR5 and CXCR4) were expressed in 2.6 and 10.1% of the neutrophil population, respectively. This demonstrates that the expression profile for HIV receptors is different between blood and genital neutrophils, with higher expression in the genital mucosa.

Next, we investigated HIV internalization through endocytosis. We first measured the expression of Rab5 after HIV stimulation. Rab5 is involved in the formation of the early endosome (55), which has been reported to be important for HIV endocytosis in cells with low or absent expression of HIV receptors (56). Fluorescence microscopy revealed Rab5 expression in a small number of blood neutrophils, which co-stained with HIV-GFP (**Figures 5C, D**). We next visualized Rab5 in genital tissues and identified a small number of genital neutrophils expressing Rab5 (**Figure 5E**). Quantification of Rab5 positive cells in multiple fields from multiple patients (blood: n=6; FGT: n=6) revealed a significant increase in Rab5+ cells after HIV stimulation in blood neutrophils (at 5 min) and in genital mixed cell suspension (at 15 min) (**Figure 5F**).

To determine if viral endocytosis was involved in NET release, we blocked dynamin-dependent internalization, previously described to be implicated in HIV endocytosis in human placental cells (56). Blood neutrophils and genital mixed cell suspensions were incubated with Dynasore, a dynamin inhibitor, prior to HIV stimulation and NET release quantification. Dynamin inhibition significantly reduced NET release in blood neutrophils (77% median reduction; p=0.03), and in genital neutrophils (31% median reduction: p=0.05) after HIV stimulation (**Figure 5G**).

These results indicate that dynamin-dependent endocytosis of HIV by neutrophils is partly implicated in NET release. Further, we demonstrate that a small proportion of neutrophils express HIV receptors, particularly in the FGT.

### Annexin V and lactoferrin identify proinflammatory NETs produced after HIV recognition by TLR7

**Figure 6:**
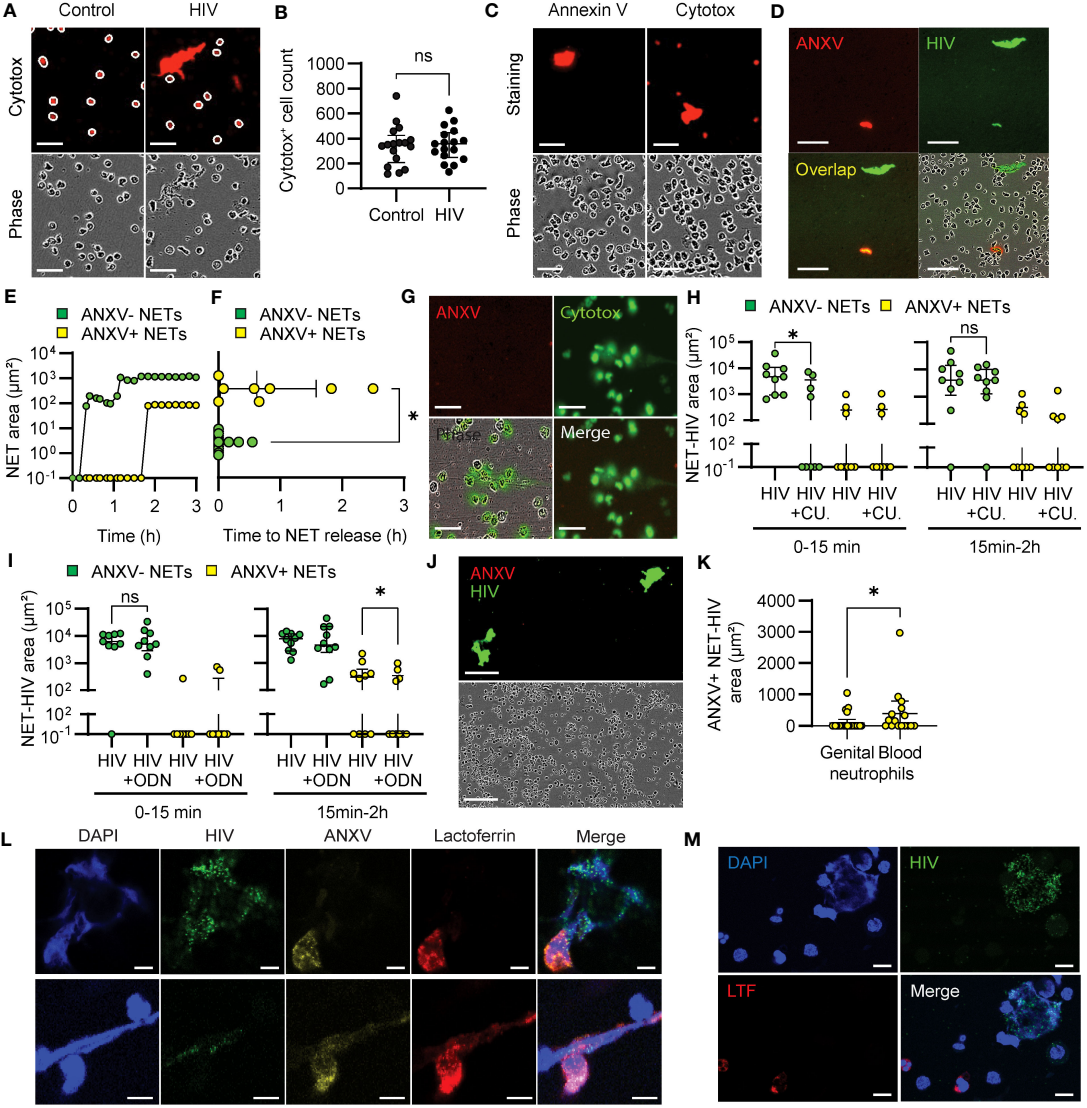
Annexin V identifies proinflammatory NETs. **(A)** Representative images of blood neutrophils stained with red cytotox in resting and HIV-stimulated conditions (30 minutes). White circles show the mask applied to quantify cytotox^+^ cells. Scale bar: 50 μm. **(B)** Quantification of cytotox^+^ blood neutrophils in control and HIV-stimulated conditions at 30 minutes. **(C)** Representative images of HIV-stimulated blood neutrophils stained with annexin V or cytotox (30 minutes). Scale bar: 50 μm. **(D)** Representative images of annexin V+ and annexin V- NETs after HIV stimulation of blood neutrophils. scale bar: 100 μm. **(E)** Representative curve and **(F)** quantification of annexin V+ (yellow) and annexin V- (green) NET production by blood neutrophils after HIV stimulation. Wilcoxon test. **(G)** Representative images of blood neutrophils stimulated with calcium ionophore in the presence of green cytotox and red annexin V (3 h). Scale bar: 50 μm. **(H, I)** Annexin V+ and annexin V- NET production after HIV stimulation of blood neutrophils preincubated with TLR8 inhibitor **(H)** or TLR7 inhibitor **(I)**. Wilcoxon test. **(J)** Representative images of the lack of annexin V+ NET production by genital neutrophils. Scale bar: 150 μm **(K)** Comparison of annexin V+ NET production by blood and genital neutrophils. Mann-Whitney test; **(L, M)** Representative confocal microscopy images of annexin V and lactoferrin expression in NETs from blood neutrophils **(L)** (Scale bar: 5 μm), and genital neutrophils **(M)** following 30min-2h stimulation with HIV (HIV-GFP, green). Scale bar: 10 μm. *p<0.05; ns, not significant..

Two main mechanisms of NET formation have been described: lytic NET release, a NADPH ROS-dependent delayed process that implicates cell membrane rupture and death; and rapid non-lytic NET release, independent from ROS production and after which neutrophils remain viable (30, 31). Since we observed that HIV exposure induced early and late NET-release through different ROS-dependent and independent pathways, we next investigated whether cell death could be involved in late NET-release in response to HIV stimulation. The reagent we used to label extracellular DNA in NETs (cytotox) also stains intracellular DNA when the cell membrane is compromised, and it is generally used to identify dead or dying cells. Therefore, to quantify dead cells, we counted the number of cytotox+ neutrophils following HIV stimulation as round intracellular red staining and excluded NETs, which are large, irregular, extracellular objects (**Figure 6A**). We found no differences in the number of cytotox+ neutrophils cells in control and HIV conditions (**Figures 6A, B**).

To determine if there was a difference in the form of death in cytotox+ neutrophils from control and HIV-stimulated conditions, we extended our study to investigate apoptosis by adding annexin V to the media prior to HIV stimulation. We did not detect annexin V+ staining in neutrophils, even when they were cytotox^+^ (**Figure 6C**), indicating that neutrophils were not undergoing apoptosis. Interestingly, we observed differential annexin V incorporation into a subset of NETs in response to HIV stimulation (**Figure 6D**). Kinetic analysis of NET formation revealed that annexin V+ NETs were released at later time points compared to annexin V- NETs (**Figure 6E**, **Supplementary Movie 6**). Quantification of the first annexin V+ and annexin V- NET released in blood neutrophils revealed a significant delay in the formation of annexin V+ NETs (**Figure 6F**). Because lytic NET formation implies the disruption of the cell membrane, while non-lytic NET release maintains cell membrane integrity (30), we hypothesized that annexin V was incorporated in NETs released through lytic mechanisms. As a control, we used calcium ionophore, known to induce non-lytic NET release through calcium mediated pathways (33). As seen in **Figure 6G**, calcium ionophore stimulation did not induce annexin V+ NETs, suggesting that annexin V+ is incorporated into NETs released after lytic mechanisms.

Next, we investigated whether annexin V+ NET release was dependent on TLR stimulation. Since annexin V+ NETs were released at later time points, we speculated that their induction could be dependent on TLR7 stimulation. As seen in **Figure 6H**, TLR8 inhibition (CU-CPT9a) significantly reduced early release of annexin V- NETs but had not effect on annexin V+ NET release. In contrast, TLR7 (ODN 20958) inhibition significantly reduced annexin V+ NETs, with no effect on annexin V- NETs (**Figure 6I**). These results suggest that TLR8 activates non-lytic NET release while TLR7 is involved in the lytic and ROS-dependent NET release pathways.

As part of these studies, we investigated the release of annexin V+ NETs by genital neutrophils. Interestingly, genital neutrophils rarely produced annexin V+ NETs (4 out 26 tissue samples) and this production was significantly lower compared to blood neutrophils (8 out 16 samples) (**Figures 6J, K**), suggesting preferential non-lytic NET release by tissue neutrophils.

Because lytic NET release has been described as proinflammatory, we explored potential functional differences between annexin V+ and annexin V- NETs by analyzing incorporation of lactoferrin (LTF), known for its proinflammatory and chemoattractant properties (57). We stimulated blood and genital neutrophils with HIV and determined incorporation of annexin V and LTF into NETs using confocal microscopy. In blood neutrophils we observed co-expression of annexin V and LTF in some NETs, while annexin V- NETs did not incorporate LTF (**Figure 6L**). In genital neutrophils, LTF was detected inside the neutrophils, but was not incorporated into NETs (**Figure 6M**). These data suggest a proinflammatory role for annexin V+ NETs.

Overall, these results demonstrate that blood and genital neutrophils release NETs with a different protein composition in response to HIV, and that annexin V and LTF can be used as markers to visualize NETs formed by lytic pathways.

## Discussion

Our study demonstrates that as women age, neutrophil responses to HIV stimulation are impaired, resulting in delayed release of NETs by neutrophils in peripheral blood and genital tissues. We demonstrate that HIV activates differential pathways in blood and genital neutrophils for the release of NETs, with preferential calcium-dependent triggering mechanisms in peripheral blood, but predominant endosomal TLR recognition in genital tissue neutrophils. This differential signaling results in distinct underlying mechanisms that reduce NET formation in blood and genital tissues with aging. In blood neutrophils, HIV fails to induce rapid calcium responses in older women, while in genital tissues TLR signaling is progressively down-regulated as women age. Importantly, our results demonstrate that blood and genital neutrophils release NETs with distinct functional activity, and we identify a subset of potentially proinflammatory NETs that incorporate annexin V and lactoferrin and are primarily produced by blood neutrophils. Our results demonstrate, for the first-time, compartment-specific and age-specific control of NET release in response to HIV and provide mechanistic insight for age-dependent functional impairment of anti-HIV responses, as well as identification of novel markers to discriminate between lytic pathological proinflammatory NETs and non-lytic protective NETs.

We previously demonstrated that genital neutrophils release NETs in response to HIV, minutes after HIV stimulation, and NETs inactivate the virus preventing infection of HIV-target cells (37). Viral inactivation and entrapment have also been described for blood neutrophils by others and us (36, 37). However, the underlying mechanisms that trigger NET release in response to HIV infection by blood and genital neutrophils and whether this anti-HIV defense mechanism is affected by aging remained unknown. Here we found a delayed anti-HIV response in neutrophils in postmenopausal women in blood and genital tissues. Menopause is a determining event in the aging process of women where there is a pronounced decrease in sexual hormones. However, although premenopausal women showed significantly higher levels of E2 in plasma compared to postmenopausal women, there was no significant correlation between HIV-NETs and E2 levels, indicating that the decrease of estrogen levels in postmenopausal women is not directly affecting the release of HIV-induced NETs. Thus, considering that E2 decline after menopause did not directly explain changes in the release of NETs in response to HIV, we hypothesize that as women age following menopause, progressive age-related cellular senescence could be responsible for the reduction in NET release. Estrogen has been proposed to prevent cellular senescence by protecting against senescence induced by DNA damage, and by inhibiting pathways and proteins involved in the establishment of cellular senescence (58). Thus, while estrogen did not directly influence HIV-NETs release, we speculate that the observed defect in the release of NETs in response to HIV in postmenopausal women could be due to cellular senescence, a cellular state which is accelerated by reduced estrogen following menopause. Nevertheless, while we did not find a direct role for E2, it is possible that other sex hormones also reduced after menopause, such as progesterone, may play a role in controlling NET release. Future studies are needed to address this gap in knowledge.

In the context of HIV prevention, this delayed response suggests that older women would be unable to immediately respond to viral challenge, potentially contributing to increased susceptibility to infection in the FGT. Whether reduced NET release would result in reduced HIV entrapment and enhanced free HIV particles able to infect target cells remains to be determined. Interestingly, we observed that genital neutrophils from the endometrium significantly increased NET release in the perimenopausal years (39), indicating compartment specific differences within the female reproductive tract that deserve further investigation.

Importantly, we identified differences in how aging impairs NET release in blood and genital neutrophils in response to HIV. In the genital tract, TLR signaling was the main pathway involved in HIV-induced NET-release in premenopausal women, and TLR8-mediated viral recognition (but not TLR7) was progressively reduced as women age, resulting in a specific impairment of early NET release. In contrast, in blood neutrophils from postmenopausal women TLR8 and TLR7 signaling remained functionally conserved. While prior work demonstrated that the TLR7/8 pathway is implicated in late NET release in response to HIV in blood neutrophils (36), and in genital neutrophils (37), here we expand these findings by uncovering a sequential involvement of TLR8 and TLR7 for early and late NET-release respectively within 2h of HIV stimulation in blood and genital neutrophils. Further, we demonstrate that TLR8 is responsible for initiating NET formation in genital neutrophils and blood neutrophils from older women. The reason for preferential TLR8 signaling remains to be determined, but it may be related to enhanced preferential TLR8 expression over TLR7 in neutrophils (52).

Another novel finding in our study is the demonstration that HIV induces a rapid intracellular calcium response that triggers NET release in blood neutrophils from younger women, but this response is completely absent in genital neutrophils, uncovering a dramatic difference in the mechanisms responsible for NET formation in blood and genital neutrophils. Importantly, endosomal TLR-mediated NET release and calcium-mediated NET release in response to HIV are two parallel and independent pathways since intracellular calcium levels remained unaltered when TLR-signaling was blocked prior to HIV stimulation. Our data indicate that TLR-mediated NET release was dependent on viral endocytosis, through a Rab5 and dynamin dependent mechanism, while intracellular calcium responses were triggered by the HIV envelope, suggesting a role for the HIV receptors CD4 and CCR5 in this process. Even though neutrophils are not considered HIV target cells, our results demonstrate HIV-receptor and co-receptor expression on blood neutrophils, although at very low levels, consistent with prior studies (38, 53, 59). Interestingly, even though we observed higher expression of HIV-receptors on genital neutrophils than blood neutrophils, HIV stimulation did not induce calcium responses in neutrophils from genital tissues, suggesting the presence of mechanisms that actively suppress calcium responses to HIV in genital neutrophils. Further studies are needed to decipher the molecular mechanisms responsible for calcium release after HIV stimulation. Importantly, in some viral infections, such as hepatitis B virus (HBV) or human herpes simplex viruses (HSV), intracellular calcium increase results in regulation of innate immune responses and transcription factors that help support viral replication cycle (60). Whether HIV-induced calcium increase in blood neutrophils plays a protective role or is involved in viral pathogenesis in peripheral blood remains to be investigated.

Two main types of NET release have been described: lytic, a ROS-dependent mechanism of late NET release that involves cell membrane disruption, and non-lytic NET release, a rapid mechanism in which NETs are extruded from the cell and neutrophils remain viable. Lytic NETs promote a proinflammatory environment and have been implicated in pathological conditions of sterile inflammation and bacterial infections (31), while non-lytic NETs are thought as antimicrobial and released in response to bacterial and fungal pathogens (30, 31). To date, no reliable markers exist to discriminate between these two types of NETs. Here we identified two types of NETs released in response to HIV based on the incorporation of annexin V with differential temporal and functional properties. Annexin V+ NETs were released at later time points and were functionally distinct from annexin V- NETs by the specific incorporation of lactoferrin, a proinflammatory molecule previously described to be secreted by apoptotic cells (61). Lactoferrin promotes monocyte and macrophage chemoattraction, while inhibiting the migration of neutrophils and eosinophils (57). We speculate that annexin V+ NETs originate from neutrophils that disrupt their membrane to release NETs, exposing phosphatidylserine, which is located in the internal leaflet of the plasma membrane and binds to annexin V after membrane disruption (30, 31). We therefore propose that annexin V incorporation into NETs is a potential marker to visualize lytic NET formation with proinflammatory or pathological potential. The distinction between the two mechanisms of NET release is relevant because NETs have been described as a double-edged sword: protective due to their antimicrobial activity, but also a source of pathological inflammation that promotes tissue damage (62). Importantly, the majority of NETs released in response to HIV stimulation were annexin V-, suggesting a preferential non-lytic mechanism. Annexin V+ NETs were released primarily by HIV-stimulated blood neutrophils, but not genital neutrophils, suggesting that the induction of NET release by HIV in the female genital tract would not promote tissue inflammation and recruitment of HIV-target cells. The tissue environment mechanisms that prevent the formation of NETs with tissue damage potential deserves further investigation.

An important limitation of our study is that while we used R5 tropic HIV strains to study molecular mechanisms of HIV recognition by neutrophils, we were not able to test any transmitted-founder (TF) HIV strains (i.e. those HIV strains that have successfully established clinical infection in a new host individual). Whether TF HIV strains induce the release of NETs and are sensitive to inactivation by NETs is a very important aspect that remains to be determined in future studies discerning HIV transmission mechanism. Nevertheless, our study defining the molecular mechanisms involved in HIV recognition by neutrophils and NET release in response to R5 tropic HIV strains that are not TF is still relevant, as it may represent a mechanism by which R5 tropic HIV-1 strains are inactivated in the genital tract.

Overall, our results demonstrate that the release of NETs in response to HIV stimulation is delayed and progressively declines in blood and genital neutrophils as women age. We identify parallel independent compartment-specific and age-dependent mechanisms that induce NET formation through HIV-RNA recognition by endosomal TLRs as well as rapid intracellular calcium increase. We speculate that multiple pathways are activated in parallel at the same time to allow NET release, suggesting that this mechanism is important for HIV inactivation. Importantly, we identified markers that discriminate proinflammatory NETs and demonstrate that proinflammatory NETs are rarely released by genital neutrophils, suggesting low tissue damage and minimal HIV-target cell recruitment potential in response to HIV stimulation at the female genital tract, a main portal of entry for HIV (37). Importantly, the impairment of this antiviral mechanism in women as they age may contribute to their increased susceptibility to HIV acquisition.

## Data availability statement

The original contributions presented in the study are included in the article/**Supplementary Material**. Further inquiries can be directed to the corresponding author.

## Ethics statement

The studies involving humans were approved by Health Sciences Institutional Review Board at Tufts University. The studies were conducted in accordance with the local legislation and institutional requirements. The participants provided their written informed consent to participate in this study.

## Author contributions

LML: Conceptualization, Data curation, Formal analysis, Investigation, Methodology, Visualization, Writing – original draft, Writing – review & editing. AW: Formal analysis, Investigation, Visualization. AB: Formal analysis, Investigation, Visualization. FCS: Investigation, Visualization. WM: Investigation, Visualization. SP: Investigation. VI: Resources. AV: Resources. DI: Resources. ACAM: Validation. CO: Investigation, Resources, Validation, Writing – review & editing. CRW: Validation, Writing – review & editing. MRG: Conceptualization, Data curation, Funding acquisition, Investigation, Methodology, Project administration, Validation, Writing – review & editing.
