# Supplementary material for: Aging dysregulates neutrophil extracellular trap formation in response to HIV in blood and genital tissues
Source: Front Immunol. 2023 Nov 15;14:1256182. doi: 10.3389/fimmu.2023.1256182 (PMC10684664; doi:10.3389/fimmu.2023.1256182)
Supplement: Supplementary file 7 [file DataSheet_1.docx]

**Supplementary material**

**Figure S1**


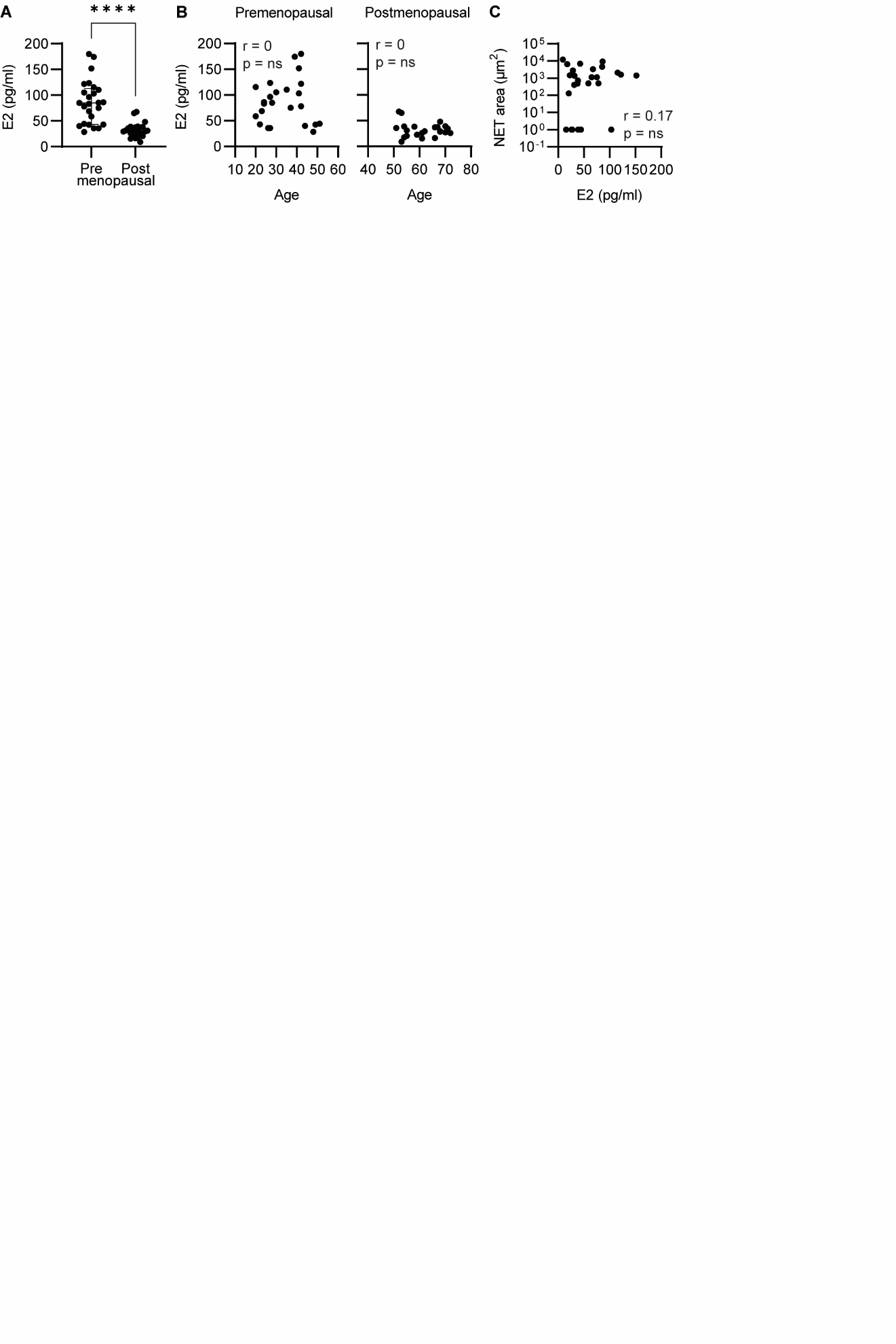


**Supplementary Figure 1: Estradiol levels in plasma from pre and postmenopausal women.** **(A)** Comparison of estradiol levels in plasma between premenopausal (n=25) and postmenopausal (n=24) women. Mann-Whitney test. ****p<0.0001. **(B)** Correlation between estradiol levels and age in premenopausal women (left graph) and postmenopausal women (right graph). Spearman r correlation (two-tailed). **(C)** Correlation between estradiol plasma levels and early NET release (15 minutes) after stimulating purified blood neutrophils from pre and postmenopausal women with HIV. Values of 0 were replaced by 1. Spearman r correlation (two-tailed).

**Figure S2**


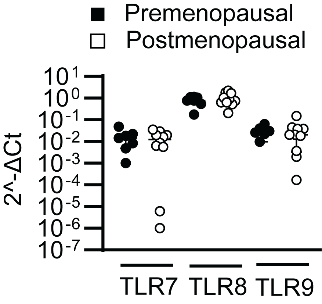


**Supplementary Figure 2: Endosomal TLR expression in blood neutrophils from premenopausal and postmenopausal women.** Expression of TLR7, TLR8, and TLR9 as determined by RT-PCR in neutrophils from premenopausal (n=7; black) and postmenopausal (n=10; white) women.

**Supplementary Movie 1:** TLR8 inhibition blocks HIV-induced NETs in blood neutrophils at early time points.

**Supplementary Movie 2:** TLR7/9 inhibition blocks HIV-induced NETs in blood neutrophils at late time points.

**Supplementary Movie 3:** Blood neutrophils rapidly increase intracellular calcium after HIV stimulation.

**Supplementary Movie 4:** Lack of intracellular calcium response in genital neutrophils after HIV stimulation.

**Supplementary Movie 5:** Blood neutrophils increase ROS production after HIV stimulation.

**Supplementary Movie 6:** Annexin V- NETs are released at earlier time points than annexin V+ NETs.
